# Structural Changes of Sarco/Endoplasmic Reticulum Ca^2+^-ATPase Induced by Rutin Arachidonate: A Molecular Dynamics Study

**DOI:** 10.3390/biom10020214

**Published:** 2020-02-01

**Authors:** Yoel Rodríguez, Magdaléna Májeková

**Affiliations:** 1 Department of Natural Sciences, Eugenio María de Hostos Community College of The City University of New York, 500 Grand Concourse, Bronx, New York, NY 10451, USA; yrodriguez@hostos.cuny.edu or; 2 Department of Pharmacological Sciences, Icahn School of Medicine at Mount Sinai, 1425 Madison Avenue, New York, NY 10029, USA; 3 Center of Experimental Medicine of Slovak Academy of Sciences, Institute of Experimental Pharmacology and Toxicology, Department of Biochemical Pharmacology, Dubravska cesta 9, 841 04 Bratislava, Slovakia

**Keywords:** rutin derivatives, SERCA1a, inhibition, molecular dynamics, calcium transport, proton transport

## Abstract

Sarco/endoplasmic reticulum Ca^2+^-ATPase (SERCA) maintains the level of calcium concentration in cells by pumping calcium ions from the cytoplasm to the lumen while undergoing substantial conformational changes, which can be stabilized or prevented by various compounds. Here we attempted to clarify the molecular mechanism of action of new inhibitor rutin arachidonate, one of the series of the acylated rutin derivatives. We performed molecular dynamics simulations of SERCA1a protein bound to rutin arachidonate positioned in a pure dipalmitoylphosphatidylcholine bilayer membrane. Our study predicted the molecular basis for the binding of rutin arachidonate towards SERCA1a in the vicinity of the binding site of calcium ions and near the location of the well-known inhibitor thapsigargin. The stable hydrogen bond between Glu771 and rutin arachidonate plays a key role in the binding. SERCA1a is kept in the E2 conformation preventing the formation of important salt bridges between the side chains of several residues, primarily Glu90 and Lys297. All in all, the structural changes induced by the binding of rutin arachidonate to SERCA1a may shift proton balance near the titrable residues Glu771 and Glu309 into neutral species, hence preventing the binding of calcium ions to the transmembrane binding sites and thus affecting calcium homeostasis. Our results could lead towards the design of new types of inhibitors, potential drug candidates for cancer treatment, which could be anchored to the transmembrane region of SERCA1a by a lipophilic fatty acid group.

## 1. Introduction

Calcium signaling plays an important role in many physiological processes, such as muscle contraction, gene expression, cell motility, apoptosis, and insulin secretion [1,2,3,4]. A primary role in the maintenance of intracellular Ca^2+^ concentration belongs to SERCA—sarco/endoplasmic reticulum Ca^2+^-ATPase, which pumps calcium ions against a concentration gradient from the cytoplasm to the luminal space of the sarco/endoplasmic reticulum. The energy necessary for active transport comes from adenosine triphosphate (ATP) hydrolysis occurring in the cytoplasmic part of SERCA. An impaired function of Ca^2+^-ATPase has been observed in connection to various chronic diseases and disorders, such as cardiovascular diseases, neurodegenerative and muscular disorders, inflammation, diabetes, and cancer [5,6,7,8]. Due to the large number of calcium pump diverse roles, SERCA inhibitors (i.e., decreasing activity of SERCA can promote apoptosis of cancer cells), as well as activators (i.e., activation is desirable when SERCA is down-regulated, or its activity is reduced by certain disorders; e.g., in cardiomyocytes of diabetic patients), are interesting for drug development.

SERCA is a member of P-type ATPases family, together with Na^+^/K^+^-ATPase, H^+^-ATPase, and H^+^/K^+^-ATPase. It occurs in several isoforms including:

SERCA1—located in the sarcoplasmic or endoplasmic reticula of fast-twitch skeletal muscle cells and plays a key role in muscle contraction. This isoform has two subforms, SERCA1a (the adult form) and SERCA1b (the neonatal form) with the last eight amino acids differing between the two ones [9];

SERCA2a—in cardiac or slow-twitch skeletal muscles and brain;

SERCA2b—in vascular smooth muscles and most other tissues; and

SERCA3a—distributed in vascular endothelium, tracheal epithelium, mast cells, and lymphoid cells [10,11].

SERCA1a is an isoform with the most thoroughly described 3D structure and structure–conditioned functional changes. There are two basic types of SERCA1a structure, E2 and E1 [12], with a lot of structural data measured using various ligands and under different acidic conditions. The reaction diagram comprising the most important catalytic steps representing structural states has been stated in detail by Toyoshima et al., 2013 [13].

Inhibitors of SERCA, and especially of SERCA1a, are formed by scaffold diversity compounds, which are classified according to their structure and molecular mechanism of action. Besides the endogenous compounds phospholamban and sarcolipin, which participate in the regulation of SERCA activity [14,15], several natural compounds and their derivatives have been found to be able to inhibit the activity of calcium pump [16,17]. Thapsigargin (sesquiterpene lactone isolated from *Thapsia garganica*) is the strongest inhibitor known so far, inhibiting SERCA irreversibly in the nanomolar region [18,19]. Cyclopiazonic acid is a toxic fungal secondary metabolite, whose toxicity is related to its potency to inhibit SERCA [20]. In addition, it has been shown that polyphenolic compounds quercetin, curcumin and galangin inhibit SERCA in the low micromolar region [21,22].

Further intensive screening has also revealed SERCA inhibition activity among compounds of synthetic origin, such as clotrimazol (antifungal drug) [23], tetrabromobisphenol A and hexabromocyclododecane (flame retardants used in polymers) [24,25], 1,5-ditert-butylhydroquinone (food preservative) [26], chlorpromazine and fluphenazine (antipsychotic drugs) [27]) and others [16]. According to the physicochemical properties, inhibitors can stabilize SERCA either in E1- or in E2-like conformation, which corresponds to their interaction with either the cytosolic or transmembrane portion of SERCA, respectively (see Table 1 in reference [16]).

**Figure 1:**
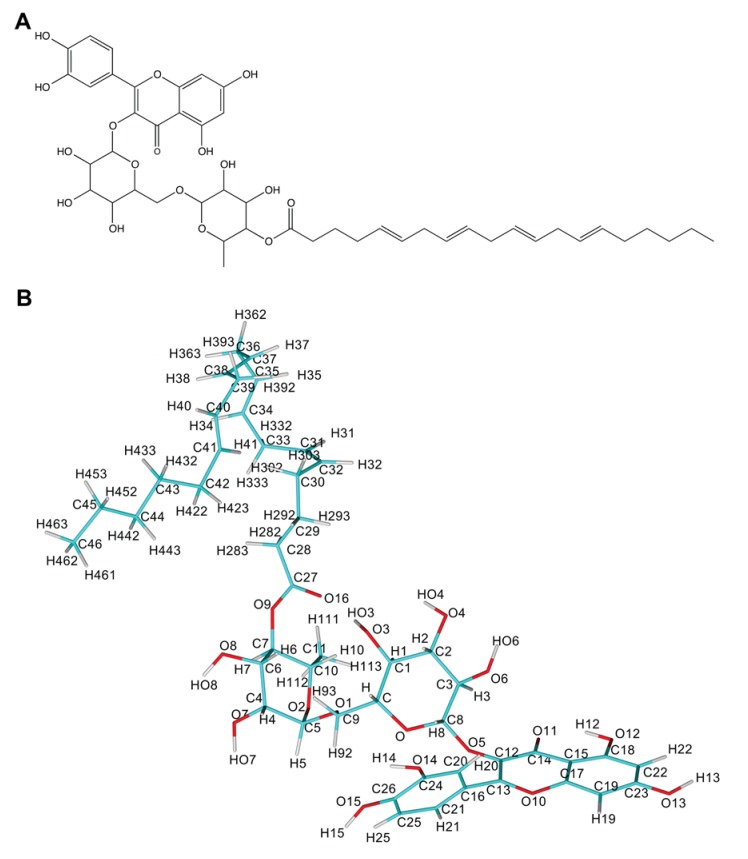
Rutin arachidonate (**A**) 2D structure; (**B**) atom names. Panels (A) and (B) were rendered using ChemDraw and YASARA [42] programs.

Recently, a series of rutin derivatives was prepared by using lipase-catalyzed acylation of rutin with aliphatic fatty acids of different chain lengths and different numbers of double bonds [28,29]. Acylation of flavonoids also occurs naturally in plants using various kinds of acyltransferases. However, the enzymatic synthesis of acylated flavonoids [30] came into attention as the most efficient way on how to obtain larger amounts of these compounds. Acylation brings numerous benefits for chemical and biological properties when comparing them with original flavonoids [31,32]. Six of the acylated rutin derivatives tested experimentally revealed their ability to inhibit the activity of SERCA1a from rabbit fast-twitch skeletal muscle [33] where rutin arachidonate (RA) (Figure 1) was the most potent inhibitor.

On the other hand, although the SERCA1a system has been extensively studied experimentally, to the best of our knowledge, few molecular dynamics (MD) simulation studies targeting this protein have been performed. Among them, we found the work by Sonntag et al., 2011 [34] where the SERCA1a-lipid interactions were analyzed using MD simulations. They found that SERCA1a adapts to different hydrophobic thickness membranes by inducing local deformations in the lipid bilayers and by undergoing small rearrangements of the amino-acid side chains and helix tilts. Recently, another MD simulation study aimed at examining the effects of nonannular lipid binding on the stability of the calcium pump SERCA1a has been conducted [35]. They found that the structural integrity and stability of SERCA1a E2 state is independent of nonannular lipid binding. A great deal of attention has also been paid to the interaction of SERCA1a with its endogenous modulators phospholamban [36,37] and sarcolipin [38]. Other MD simulation studies have dealt with the protonation and proton transfer in SERCA1a [39,40,41].

As the experimentally tested rutin derivatives brought new structural features (e.g., long fatty acid chain) into the diversity of SERCA inhibitors as described above, we reasoned to perform a molecular dynamics simulation study to elucidate the mechanism of action of the most effective inhibitor of the series, rutin arachidonate [33] (see Figure 1). Our results predicted that the binding of rutin arachidonate towards SERCA1a occurs in the vicinity of the binding site of calcium ions (transmembrane region) and near the location of the well-known inhibitor thapsigargin. Rutin arachidonate engages in a stable hydrogen bond with Glu771 in SERCA1a, playing a key role in the binding. In addition, SERCA1a remains in the E2 conformation avoiding the formation of important salt bridges, such as the one between Glu90 and Lys297. This study could help in designing novel inhibitors targeting the transmembrane region of SERCA1a by incorporating a lipophilic fatty acid group.

## 2. Materials and Methods

### 2.1. Bilayer Setup

The X-ray structure of SERCA1a corresponding to the E2 state was used as a starting protein structure (PDB ID 3w5c). The structured potassium ion was kept and used for further system setup. The protonation states of the residues forming the transport site were modeled as follows: Glu309, Glu771, and Glu908 were protonated, while Asp800 was ionized as done in previous studies of the E2 intermediate state [35,41]. A disulfide bond was created between Cys876 and Cys888. The rutin arachidonate ligand had previously been docked against SERCA1a using YASARA (www.yasara.org) [42]. As a result, a complex model of SERCA1a-RA was built and used as a starting structure to construct the binary system [33]. We generated the binary system model with pure 1-palmitoyl-2-oleoyl-sn glycero-3-PC (POPC). The starting bilayer system model was constructed using the CHARMM-GUI Membrane Builder [43] and converted to Lipid14 PDB format using the charmmlipid2amber.x script available in AmberTools14 [44]. The protein structure was initially oriented in the membrane, as proposed by the Orientations of Proteins in Membranes (OPM) database (https://opm.phar.umich.edu) [45]. The bilayer system model was built in a rectangular box containing a total of 402 lipids (202 on the upper leaflet and 200 on the bottom leaflet). The SERCA1a-RA-POPC bilayer system was solvated using TIP3 water molecules to make a simulation box with dimensions 121 × 121 × 172Å^3^. The overall charge of the system was neutralized with K^+^ and Cl^−^ ions yielding an ionic concentration of 150 mM. The hydrogen bond energies were calculated by using the ListHBoMol command in the YASARA program with the default value of 6.25 kJ/mol for minimum energy required to list a hydrogen bond. The resulting values (depending on hydrogen-acceptor distance and bond angle depending on scaling factors) are positive due to the YASARA convention, where the bond energy is by definition the energy required to break the bond. Amber14 force field was used for the calculations of the hydrogen bond energies. The hydrophobic and π-π interactions (calculated by using the ListIntMol command in YASARA) were searched using a cutoff distance of 4.0 Å between the hydrophobic atoms and no occlusion accepted. Then, the interaction strength between 0 (detectable) and 1 (optimal) was assigned to each of the hydrophobic and π-π interactions in YASARA.

### 2.2. Molecular Dynamics Simulation Protocol

We performed a 200 ns molecular dynamics simulation of the SERCA1a-RA-POPC bilayer system model using the AMBER 14 package. The all-atom FF14SB and Lipid14 Amber force fields [44,46,47,48] and the TIP3P water molecules model [49] were used for the system calculations. The system was initially minimized for 10,000 steps using the steepest descent (5000 steps) and conjugate gradient (5000 steps) methods to remove unfavorable steric interactions. Weak restraints on the protein, including the structured potassium ion (force constant 10 kcal mol^−1^ Å^−2^) and POPC lipid (force constant 2.5 kcal mol^−1^ Å^−2^), were imposed. Then, the system was heated at a constant rate to 310 K using Langevin dynamics [50] for 25 ps at constant volume, with weak positional restraints on the protein including the structured potassium ion, the ligand (force constant 10 kcal mol^−1^ Å^−2^) and lipid molecules (force constant 2.5 kcal mol^−1^ Å^−2^). Afterward, the volume was allowed to change freely, and the temperature was kept constant with a Langevin collision frequency of γ = 1.0 ps^−1^ and anisotropic Berendsen regulation [51] (1 atm) with a Berendsen coupling constant of taup = 0.5 ps. The positional restraints on the protein, ligand, and lipid molecules were gradually reduced from 10 kcal mol^−1^ Å^−2^ and 2.5 kcal mol^−1^ Å^−2^ respectively, until the system was allowed to move freely. The non-bonded cutoffs of 8 Å was used for the Lennard–Jones potentials and the electrostatic interactions calculated using the particle–mesh Ewald (PME). The width of the non-bonded “skin” was set to skinnb = 5 Å. The production MD simulation was carried out using the NPT ensemble and using Particle Mesh Ewald Molecular Dynamics on GPUs (pmemd.cuda). In the production stage, the temperature was maintained at 310 K using Langevin dynamics with a collision frequency of γ = 1.0 ps^−1^. The anisotropic Berendsen regulation [51] (1 atm) with a Berendsen coupling constant of taup = 0.5 ps was also used. The length of all bonds involving hydrogen atoms was kept fixed with the SHAKE algorithm [52]. The pressure was kept fixed at 1 atm. The equations of motion were integrated with a time-step of 2 fs. The coordinates were recorded every 5 ps. All MD simulations and analyses were performed using Amber [44,46] and Simulaid [53] programs.

### 2.3. Parameterization of Rutin Arachidonate

The force field parameters for rutin arachidonate, which is neutral, were obtained by using the following protocol: (1) the geometry of the molecule, drawn using VIDA program (VIDA 4.3.0.4: OpenEye Scientific Software, Santa Fe, NM; www.eyesopen.com), was optimized using quantum mechanics semi-empirical (QMS) minimization; (2) the ligand atomic partial charge was computed with the AM1-BCC semi-empirical method as implemented in the Amber 14.0 Antechamber programs [44,46,54], and (3) the parameterization of the ligands were done using the GAFF force field [46,55].

## 3. Results and Discussion

### 3.1. Starting Structure of SERCA1a-RA Complex

**Figure 2:**
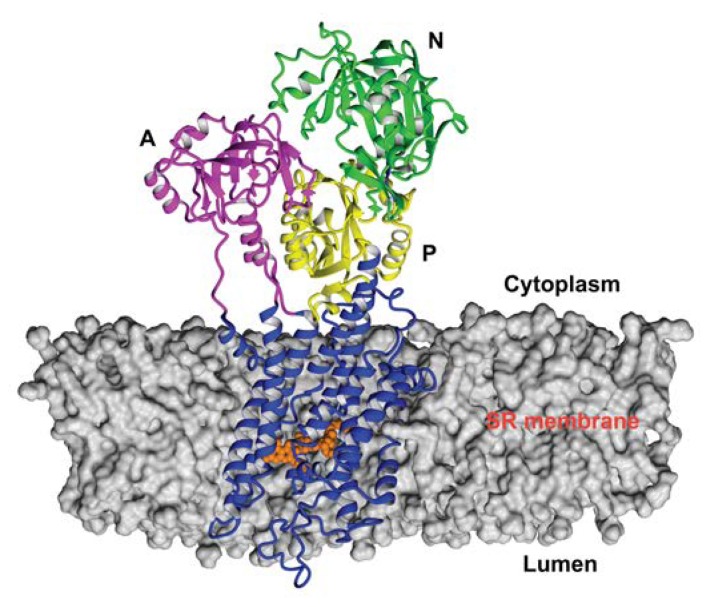
Position of RA in the 3w5c model of sarco/endoplasmic reticulum Ca^2+^-ATPase 1a (SERCA1a) obtained by docking and optimization method [33], which was the starting geometry for the MD simulation. RA molecule is shown in the ball style (orange). The A, N, and P domains in the cytoplasm are shown in pink, green, and yellow, respectively. M1 to M10 in the transmembrane region are shown in blue. Figure was rendered using YASARA [42].

In agreement with our previous results [33], the starting structure of RA complex with SERCA1a was based on the crystal structure of E2 state free from exogenous inhibitors, ATP and calcium ions (Toyoshima et al., 2013, Protein Data Bank (PDB); http://www.rcsb.org; PDB ID 3w5c). The PDB model of E2 state was chosen based on partial structural similarity with the compounds identified as SERCA1a E2 state inhibitors (thapsigargine and nonylphenol) [16], as well as according to the long lipophilic chain of RA. To test our hypothesis, we performed docking of RA against the E1 state model (PDB ID 4xou; Ca^2+^-E1-Mg-AMP-PCP) [56]. All poses found were immersed out of protein and/or showed unfavorable binding energy. The best scoring pose, obtained previously with a 3w5c structure (Viskupicova et al., 2015) [33], was found in the transmembrane part of SERCA1a (Figure 2) and stabilized by hydrogen bonds with Glu90, Glu771, Thr778, Thr848, and Lys297. The positioning of RA in the transmembrane part of SERCA1a was confirmed by using fluorescent-labeling probes as well [33]. RA significantly reduced NCD-4 binding and Trp fluorescence, which are related to the conformational changes in the transmembrane region. On the other hand, the fluorescence of FITC, linked to the cytosolic part of SERCA1a, was practically unaffected by RA [33]. In addition, it is known that the 3w5c model is a good starting point for the MD simulation of SERCA1a basic properties in the E2 state due to its high resolution of 2.5 Å [39]. All these justify the use of the calculated docked pose of RA in the SERCA1a 3w5c model as the starting geometry for the MD simulation.

### 3.2. Molecular Dynamics Simulation of SERCA1a-RA-POPC System

**Figure 3:**
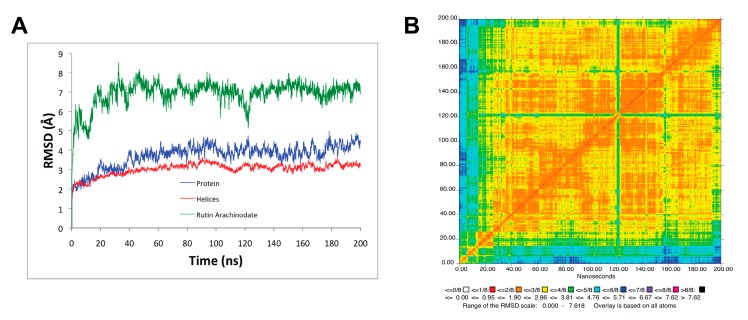
Stability of the MD simulation. (**A**) Root–mean–square deviation (RMSD) for all backbone protein, transmembrane helices, and rutin arachidonate with respect to the minimized initial structure; (**B**) 2D-RMSD for rutin arachidonate. Figure was rendered using the Simulaid program [53].

**Figure 4:**
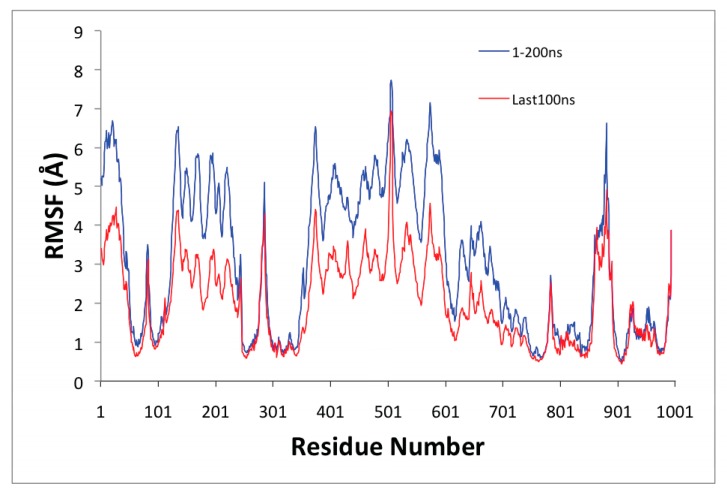
Root–mean–squared fluctuations (RMSF) values collected from the starting structure during the whole (blue) and the last 100 ns (red) molecular dynamics (MD) simulation.

We analyzed the MD simulation of the SERCA1a-RA-POPC system using the root–mean–square deviation (RMSD) (Figure 3A) and the two-dimension-RMSD (2D-RMSD; see Figure 3B), as well as root–mean–squared fluctuations (RMSF; see Figure 4).

The protein (SERCA1a) in the SERCA1a-RA complex and its transmembrane helices stabilized after 30 ns MD simulation with an average RMSD of 3.7 ± 0.6 Å and 3.0 ± 0.3 Å with respect to the minimized initial structure backbone atoms (see Figure 1A). Rutin arachidonate also stabilized from the beginning of the MD simulation with average heavy atoms RMSD of 6.9 ± 0.7 Å relative to the minimized initial structure (see Figure 3A). The 2D-RMSD also confirms the RA stabilizes beginning early in the MD simulation (see Figure 3B).

Figure 4 shows the root–mean–square fluctuations (RMSF) as a function of the residue number of the SERCA1a-RA complex. The Ca^2+^ transport site residues (Glu309, Glu771, and Glu908) displayed low mobility. The A domain (Met1 to Lys47, Ala112 to Lys246) and N domain (Thr357 to Pro602) showed high mobility as one combined body; they move together. The loop formed by residues Pro500 to Asn510 on the N domain showed high mobility along the MD simulation. Residues comprising helices M3 (Thr247 to Asn275) and M4 (Ser287 to Ile307), and the loop joining M3 and M4 formed by Ile276 to Gly286 displayed high mobility, as well. Residues forming part of helices M1 (Ser48 to Gln56), M1′ (Asp59 to Ala76), and M2 (Phe88 to Ala112) also showed high mobility. In addition, the loop formed by Tyr859 to Glu892 between helices M7 and M8 located in the lumen depicted high mobility, as well making a portion of helix M5 (Ile765 to Leu781) bend during the MD simulation. All these high mobility triggering conformational changes could, in part, explain the inhibitory effect of RA.

**Figure 5:**
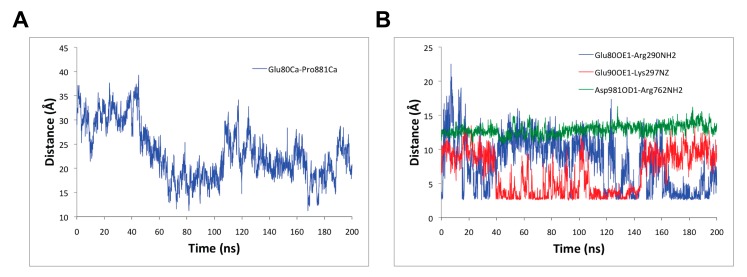
Distances (**A**) between alpha carbon atoms of Glu80 belonging to the loop joining helices M1 and M2 and Pro881 belonging to the loop joining helices M7 and M8 both in the lumen as function of time; and (**B**) between the side chain of the carboxyl oxygen OE1 of Glu80 and the –NH group of Arg290 in SERCA1a, side chain of the carboxyl oxygen OE1 of Glu90 and the –NH group of Lys297 in SERCA1a and the side chain of the carboxyl oxygen OD1 of Asp981 and the –NH group of Arg762 in SERCA1a.

In addition, to better understand the conformational changes triggered by the binding of RA to SERCA1a, several distances between key residues were measured. For example, the distance between the alpha carbon atoms of Glu80 belonging to the loop joining helices M1 and M2 and Pro881 belonging to the loop joining helices M7 and M8 both in the lumen was reduced on average in 10 Å from 31.1 ± 2.9 Å during the first 45 ns of the MD simulation to 21.0 ± 3.8 Å during the last 155 ns MD simulation (see Figure 5A). The conformational changes in the loops in the luminal part of SERCA1a have not been as extensively studied as those of other SERCA1a segments. However, Sacchetto et al., 2012 [57] have found that the Ca^2+^-ATPase of bovine muscle, which has reduced activity in comparison with the rabbit one, differs structurally from this enzyme in the M7-M8 loop conformation, which is in good agreement with our current study and the inhibitory effects of RA.

Furthermore, as shown in Figure 5B, the distances between the side chain of the carboxyl oxygen OE1 of Glu80 (of the loop joining helices M1 and M2) and the –NH group of Arg290 (in helix M4) in SERCA1a, and the side chain of the carboxyl oxygen OE1 of Glu90 (in helix M2) and the –NH group of Lys297 (in helix M4) in SERCA1a fluctuated and alternated from one state to another with average values of 6.2 ± 3.3 Å and 7.0 ± 3.0 Å, respectively, during the last 100 ns of the MD simulation. A closer analysis of these latter distances between 100 ns to 140 ns, and 140 ns to 200 ns MD simulation portions, provided average values of 7.4 ± 3.5 Å and 4.1 ± 1.8 Å, and 5.4 ± 2.8 Å and 9.1 ± 1.6 Å, respectively, which revealed the potential formation of a complementary salt bridge between either pair of residues (i.e., Glu80 - Arg290 or Glu90 - Lys297; see Figure 5B). However, the distance between the side chain of the carboxyl oxygen OD1 of Asp981 (in helix M10) and the –NH group of Arg762 (in helix M5) in SERCA1a was stable with an average value of 13.3 ± 0.7 Å, which revealed that they do not engage in salt bridge interaction. All these three distance profiles within residues in the transmembrane region were exhibited as a result of conformational changes triggered by the binding of RA to SERCA1a during the calcium transport process. As described by Chen et al., 2008 [58], the salt bridge between Arg762 and Asp981 was formed after calcium binding to site I [58]. Thus, this salt bridge was hindered by RA binding, which was confirmed by the Arg762NH - Asp981OD1 distance staying at ~13 Å during the 200 ns MD simulation (Figure 5B). On the other hand, the pairwise distances of Glu80-Arg290 and Glu90-Lys297, which were formed after Ca^2+^ binding to site II [58], alternated and complemented between two states allowing the formation and/or breaking of a salt bridge (Figure 5B). According to our calculations, RA occupied the Ca^2+^-binding site I (Glu908, Glu771 and Asp800, Asn768, Thr799 as described in [12]) instead of Ca^2+^-binding site II (Ala305, Glu309, Asn796, Asp800 as described in [12]). Thus, the presence of RA could partially promote similar changes as the substrate of the Ca^2+^-binding site I. From this point of view, RA mimics a partial competitive behavior for Ca^2+^-binding site I.

Finally, the distances among the alpha carbon atoms of the Ca^2+^-binding site residues Glu309 and Glu771 (from 12.9 ± 0.5 Å to 14.0 ± 0.4 Å), and Glu309 and Glu908 (from 16.1 ± 0.5 Å to 16.9 ± 0.5 Å) were also measured. They increased by about 1 Å by the end of the MD simulation, but the distance between Glu771 and Glu908 remained the same (12.8 ± 0.4 Å) during the MD simulation. This is in agreement with the low mobility of the Ca^2+^-binding site residues mentioned above. In this regard, it is noteworthy that the binding of RA to SERCA1a seems not to directly affect the mobility of the transport site residues, but could engage in interaction with them (see below Section 3.3).

Taken together, RA binding to SERCA1a prevents the formation of salt bridge Arg762 - Asp981 necessary for neutralization of the positively charged Arg762 residue (required for the occupancy of Ca^2+^-binding site II, as described by Chen et al., 2008 [58]) and partially restricts the formation of salt bridges Glu80-Arg290 and Glu90-Lys297 linked to the occupancy of Ca^2+^-binding site II.

### 3.3. Rutin Arachidonate Binding Site

**Figure 6:**
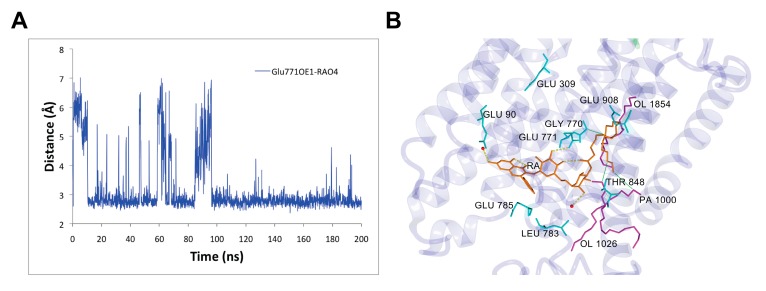
Rutin arachidonate (RA) binding site analysis. (**A**) Distance between the side chain of the carboxyl oxygen OE1 of Glu771 in SERCA1a and the hydroxyl hydrogen HO4 of RA as a function of time; (**B**) Binding mode of RA in SERCA1a. RA (in orange), residues (in cyan), and lipids (in magenta) are shown as a stick model. OL – oloeyl and PA – palmitoyl parts of 1-palmitoyl-2-oleoyl-sn glycero-3-PC (POPC). Hydrogen bonds are displayed as yellow dashed lines. Figure was rendered using YASARA [42].

We analyzed the rutin arachidonate binding site in its complex with SERCA1a. The last 100 ns MD simulation of the SERCA1a-RA complex revealed four intermittent hydrogen bonds between SERCA1a and RA with occupancy over 20% and one stable hydrogen bond with about 90% occupancy. The stable hydrogen bond was between 1) the side chain of the carboxyl oxygen of Glu771 in SERCA1a and the hydroxyl hydrogen HO4 of RA (~88% occupancy), and the intermittent ones were between 2) the carbonyl oxygen backbone of Leu783 in SERCA1a and the hydroxyl hydrogen H15 of RA (~47% occupancy); 3) the side chain of the carboxyl oxygen of Glu90 in SERCA1a and the hydroxyl hydrogen H13 of RA (~57%); 4) the side chain of the hydroxyl oxygen of Thr848 in SERCA1a and the hydroxyl hydrogen HO8 of RA (~27%); and 5) the side chain of the carboxyl oxygen of Glu785 in SERCA1a and the hydroxyl hydrogen H14 of RA (~28%) (see Table 1 and Figure 6). These latter intermittent hydrogen bonds could provide room for the design of more potent RA derivative inhibitors by making these bonds stronger.

An overall look at the RA binding site in SERCA1a revealed two different binding modes including two RA distinctive moieties: (1) The most hydrophilic flavonoid moiety of RA was bound through hydrogen bonds either to Glu771 or to surrounding water molecules, and (2) the lipidic moiety was stabilized through hydrophobic interactions to Gly770 and oloeyl or palmitoyl portions of surrounding phospholipids’ membrane (see Figure 6B).

### 3.4. Changes in SERCA1a

**Figure 7:**
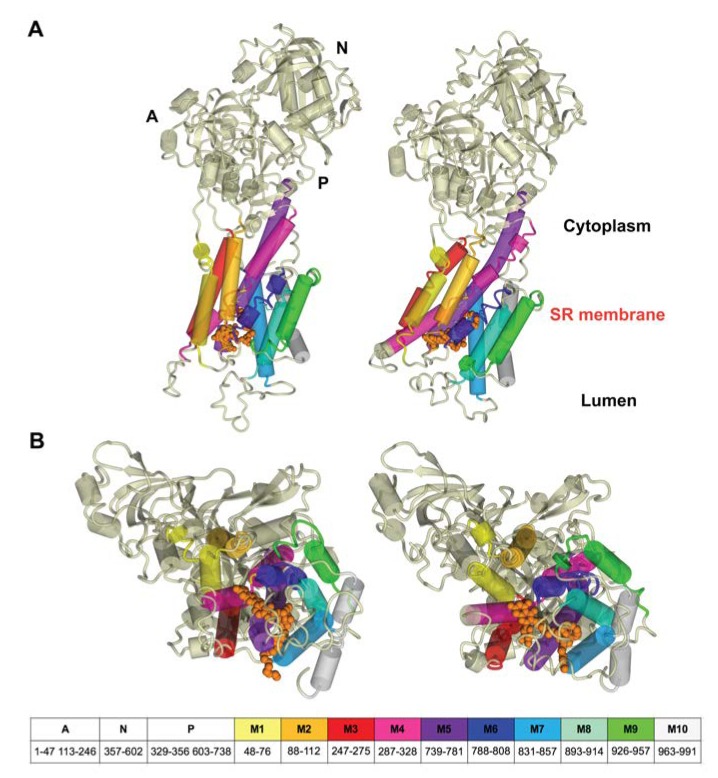
Rearrangement of transmembrane regions M1-M10 during the MD simulation: (**A**) side view; (**B**) view from the lumen. Structures at the beginning (left panel) and after 200 ns MD simulation (right panel). The cytoplasm domains (A, N, and P) and the transmembrane helices (M1 to M10) legend and residue numbering are shown at the bottom. The residues not assigned to domains are loops between individual transmembrane helices occurring in the lumen. Rutin arachidonate is depicted as a space-filling model in orange. Figures were rendered using YASARA [42].

**Figure 8:**
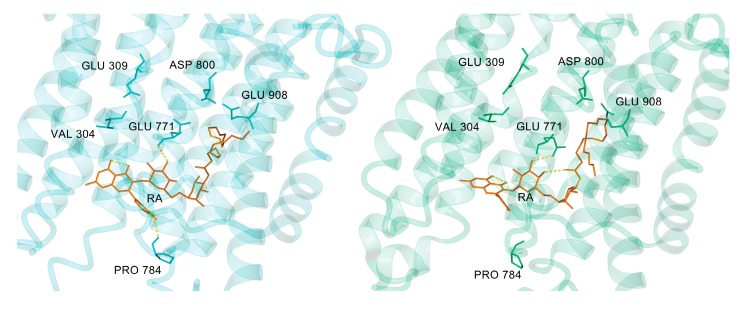
Conformation of RA (orange) and the nearby residues at the beginning (left panel; cyan) and after 200 ns (right panel; green) MD simulation. Hydrogen bonds are displayed as yellow dashed lines. An inward-to-outward carboxyl group side-chain transition of Glu309 during the MD simulation can also be seen. Figures rendered using YASARA [42].

After 200 ns of MD calculation, SERCA1a showed several structural changes as a consequence of RA binding (see Figure 7). The most striking difference was a shift of the M4-M5 helices in the transmembrane region outwards, which was linked to conformational changes in RA and its nearby residues (see Figure 8). The overall changes in the transmembrane region included two titrable residues Glu309 (gating residue; located in M4 helix) and Glu771 (in M5 helix), which played an important role in opening and closing the lumen gate for Ca^2+^ transport [41]. It is worth mentioning, as already noted in Section 3.1, that our calculations were performed using the E2 state 3w5c model, which is preferred at pH 6.5. It is known that at pH 7 and higher, the E1 state (obviously as E1·Mg^2+^ or E1·2Ca^2+^) should be the preferred one. However, currently, there is no E1 state crystallographic structure solved without ligand. In addition, Toyoshima et al., 2013 [12] have stated that “*at pH 7.5 crystals of SERCA1a grew only in the presence of 40mM MgSO_4_*”. As already mentioned above in Section 3.1, we performed docking of RA against the E1 state model (PDB ID 4xou; Ca^2+^-E1-Mg-AMP-PCP) [56] to test the suitability of the E1 state model for our modeling study. However, none of the found poses were acceptable for further modeling.

**Figure 9:**
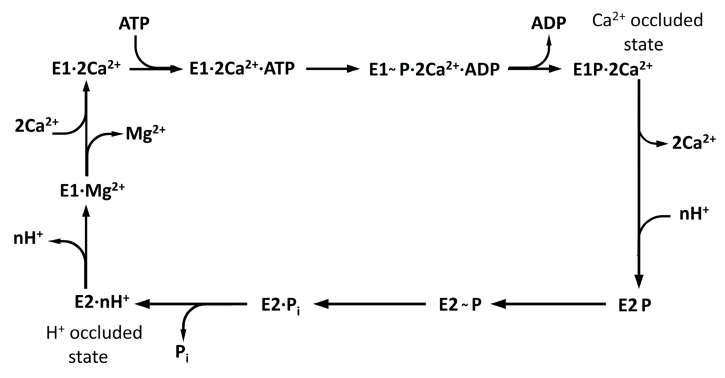
Scheme of catalytic and transport steps of the SERCA1a cycle of calcium pumping (loosely adapted from Figure 1 in reference [13]).

SERCA1a performs a complicated multistep catalytic process (see Figure 9), where individual steps depend on a lot of parameters characterizing the luminal, membrane, and cytoplasm environment (ATP, Mg^2+^, and Ca^2+^ concentrations, acidity on both sides, temperature, membrane composition) as well as regulators (sarcolipin, phospholamban). Inspecting the E2 states related to the inhibition of RA (without calcium ions inside), we can see that all states without metal ions were characterized by protons occurring in the calcium-binding sites. The rate constant of complete dephosphorylation of E2P→E2 for wild type SERCA1a was measured as k = 0.11 s^−1^. On the other hand, the rate constant of E2→2Ca^2+^E1 was estimated as almost one order higher, k = 0.9 s^−1^ [59]. That is, the rate-limiting step (or set of steps) for the second period of SERCA1a activity cycle was the E2P→E2 transition.

**Figure 10:**
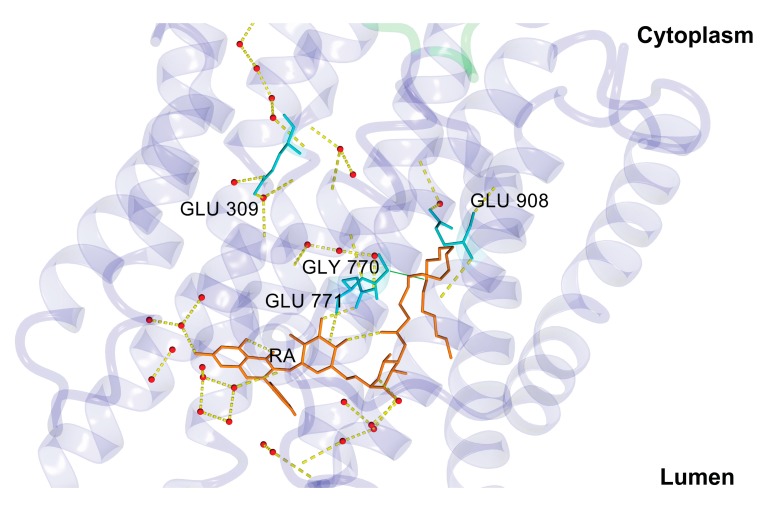
Water network for proton pathway from the lumen to the cytoplasm. Proton pathway was created, but proton transfer was not probable due to high values of pK_a_’s. RA—stick model (in orange); Ca^2+^ transport site residues—stick model (in cyan). Hydrogen bonds are displayed as yellow dashed lines. Water molecules are depicted as red spheres. Figure was rendered using YASARA [42].

The transition of the type E2-to-E1 occurred automatically at physiological pH 7.4, as without any ligand present, the titrable residues Glu309 and Glu771 became acidic [41], and under the presence of transient water clusters connecting Glu309 with the cytosol, the first proton could be transferred to the cytosol. This step was also connected to an inward-to-outward side-chain transition of Glu309, where the carboxyl group of Glu309 turned from the lumen to the cytosol orientation [41]. Surprisingly, similar structural change in Glu309 could also be observed after 200 ns of our MD simulation (see Figure 8) together with the creation of a water network (see Figure 10).

We did not include the protonation of amino acids as an implicit function for our molecular dynamics calculations in our study due to large computational demands. The protonation state of SERCA1a was analyzed before the MD simulation, and it was kept constant for the whole simulation. Nonetheless, to assess the effect of RA on the protonation state of the most important residues involved in a proton transport and consequently creating negatively charged binding cavity for Ca^2+^ ions (Glu309, Glu771, and Glu908) we calculated the pK_a_ values for these residues using PROPKA software [60,61] provided online at http://nbcr-222.ucsd.edu/pdb2pqr_2.0.0/ (see Table 2).

The first proton transport to the cytoplasm region occurred by means of Glu309 [39,41]. Under the presence of RA, the value of pK_a_ for Glu309 decreased from 9.04 to 7.31 after 200 ns of simulation. However, its average value for the whole 200 ns MD simulation (7.85 ± 0.77) was slightly higher compared to the second half of the MD simulation, which was 7.50 ± 0.34. Thus, the probability of the first proton transport was lower when compared with unsubstituted SERCA1a [39]. Moreover, according to our computational results, the second proton transport, which came from Glu771, was improbable. During the simulation time, the orientation of the side-chain carboxyl group of Glu771, which was a source of the second proton transfer, changed considerably and turned towards the RA molecule (see Figure 8) creating a stable hydrogen bond with it (see Figure 6 and Figure 8). Concurrently, under the changed surrounding conditions after 200 ns of MD, the pK_a_ value of Glu771 increased from 8.35 to 8.58. The average value for the whole MD simulation was 8.65 ± 0.51, and for the second half (100 ns–200 ns) was 8.41 ± 0.22. The third titrable residue, Glu908, increased its pK_a_ value from 10.89 to 11.71. The average value for the whole MD simulation was 10.59 ± 1.76, similar to that for the second half of the simulation (10.70 ± 1.87). Residue Glu908 is supposed to stay protonated even in the Ca^2+^-bound-E1 state [39,41]. However, this state depends strongly on the protonation of Asp800 [39]. The protonation of Asp800 immediately invokes the deprotonation of Glu908 (see Table 1 in [39]). During our simulation, the pK_a_ of Asp800 reached basic values in two cases (10.48 at 10 ns and 11.06 at 140 ns), causing a strong jump shift of the pK_a_ of Glu908 to respective acidic values (5.38 at 10 ns and 5.63 at 140 ns). The protonation of proteins is a very complex process depending on a variety of factors. We were not able to include all of them in our study, but it is also obvious that RA induces reasonable changes in the SERCA1a protonation state.

During the MD simulation, RA changed its binding mode in SERCA1a. The MD simulation started with four hydrogen bonds with residues Glu771, Pro784, Leu787, and Thr848, and three water molecules in the vicinity of the binding site (see Table 3). After 200 ns, the only hydrogen bond remaining between RA and SERCA1a was through Glu771, which was stable for the last 100 ns of the MD simulation with occupancy of ~88% (see Figure 6 and Table 1). On the other hand, the energy penalty caused by losing those three hydrogen bonds (from 69 kJ/mol to 23 kJ/mol) was replaced by a markedly stronger interaction with the water environment (total hydrogen bond energy raised from 49 kJ/mol to 88 kJ/mol; see Table 3). Analogously, the non-polar interactions became stronger during the simulation increasing the total interaction strength value (dimensionless parameter measuring the strength of hydrophobic and π-π interactions, see Table 3) from 51 to 62, underscoring the stabilizing effect of the aglycone and fatty acid chain moieties of RA keeping it (RA) inside the transmembrane region. Thus, supporting our hypothesis regarding the possible role of RA in shifting the protonation state during the MD simulation, its binding (RA) to SERCA1a strengthens the occlusion of protons in the transmembrane calcium-binding site. This occurs not only by affecting the pK_a_ values of titrable groups of residues Glu309, Glu771, and Glu908 but also by the stabilization of water networks and transmembrane helices in position stabilizing the E2 state even under higher basic conditions.

As a consequence of hindering the proton transport to the cytoplasm, SERCA1a was not able to undergo structural changes necessary for the E2-to-E1 transition, which were primarily connected to the formation of negatively charged binding cavities for Ca^2+^ ions including residues Glu309, Glu771, and Glu908. On the contrary, all three protons neutralizing these glutamic acid residues seemed to be occluded with a significant contribution of the external ligand RA.

The inhibition of SERCA, as mentioned above, is connected to the anticancer properties of various compounds. The fatty acid esters of rutin derivatives have been proved to exhibit cytotoxic and anti-proliferative activity on several cell lines (see review [31]). In this regard, it is possible that the ability to inhibit SERCA protein represents one of the molecular mechanisms of action of these compounds.

## 4. Conclusions

In this study, we used MD simulations to understand the molecular basis for the binding of rutin arachidonate towards SERCA1a embedded in a pure POPC bilayer system, as well as the mechanism underlying its effective inhibition. We used as a starting model the X-ray structure of SERCA1a (PDB ID 3w5c) in the E2 intermediate state. Our results indicated that RA binds to SERCA1a in the vicinity of the Ca^2+^-binding site I and close to the position occupied by the well-known inhibitor thapsigargin in the transmembrane region [62]. RA was engaged in a stable hydrogen bond with Glu771 playing a key role in its binding recognition by SERCA1a. The RA was also stabilized by a network of hydrophobic, π-π, and water interactions. It is worth mentioning that SERCA1a remained in the E2 intermediate state during the MD simulation avoiding the formation of key salt bridges between several residues side chains, including Arg762 and Asp981, that otherwise would enable the occupancy of Ca^2+^-binding site II of SERCA1a neutralizing the positive charge of Arg762 [58].

Altogether, we can consider rutin arachidonate to be a reversible type inhibitor of SERCA1a, keeping the protein in the E2 intermediate state by hindering the proton transport from the lumen to the cytoplasm and stabilizing the conformation of this E2 state under normal and basic conditions. This result could guide the design and development of new SERCA1a inhibitor types, possible drug candidates for the treatment of cancer, which could be anchored to the transmembrane region of SERCA1a by a lipophilic fatty acid group.

## Figures and Tables

**Table 1:** Hydrogen bonds analysis for the sarco/endoplasmic reticulum Ca^2+^-ATPase 1a–rutin arachidonate (SERCA1a-RA) complex (last 100ns).

Acceptor	DonorH	Donor	Fraction Occupied	Distance (Å)	Angle (°)
GLU771@OE1	RA995@HO4	RA995@O4	0.880	2.740	157.68
LEU783@O	RA995@H15	RA995@O15	0.472	2.739	161.95
GLU90@OE1	RA995@H13	RA995@O13	0.316	2.635	161.03
GLU90@OE2	RA995@H13	RA995@O13	0.255	2.638	160.98
THR848@OG1	RA995@HO8	RA995@O8	0.267	2.846	156.79
GLU785@OE1	RA995@H14	RA995@O14	0.216	2.625	165.96
GLU785@OE2	RA995@H14	RA995@O14	0.067	2.609	166.30
LEU783@O	RA995@H14	RA995@O14	0.109	2.681	159.78
GLU771@OE1	RA995@HO3	RA995@O3	0.061	2.814	150.23
PRO784@O	RA995@HO7	RA995@O7	0.013	2.812	157.84

Hydrogen bonds were determined via the distance between the heavy atoms using a cutoff of 3.5 Å and the angle between the acceptor and donor atoms using a cutoff of 120°. See Figure 1 for RA atom names.

**Table 2:** Mean values of predicted pK_a_ under the influence of the environment for titrable residues Glu309, Glu771, and Glu908 calculated for the whole (1–200 ns) and the second half (100–200 ns) of the MD simulation.

Simulation Time	Predicted pK_a_		
	Glu309	Glu771	Glu908
1–200 ns	7.85 ± 0.77	8.65 ± 0.51	10.59 ± 1.76
100–200 ns	7.50 ± 0.34	8.41 ± 0.22	10.70 ± 1.87

**Table 3:** Interaction parameters of the SERCA1a-RA complex with water at the beginning and after 200 ns MD simulation.

Interaction	Parameter	Simulation Time	
		1 ns	200 ns
Hbond * (SERCA1a)	*E* * (kJ/mol)	69	23
	Residues	Glu771, Pro784, Leu787, Thr848	Glu771
**Hbond (Water)**	*E* (kJ/mol)	49	88
	Number of Water Molecules	3	4
**Hydrophobic**	*Strength* *	51	61
	Number of Residues	27	29
**π-π**	*Strength*	0	1
	Number of Residues	0	3

* Hbond stands for hydrogen bond, E represents the hydrogen bond energy, and Strength is a dimensionless parameter between 0 (detectable) and 1 (optimal) that measures the strength of hydrophobic and π-π interactions. All three parameters were calculated using YASARA [42]. See Section 2.1 for further explanation.
